# Improving the prediction of patient survival with the aid of residual convolutional neural network (ResNet) in colorectal cancer with unresectable liver metastases treated with bevacizumab-based chemotherapy

**DOI:** 10.1186/s40644-024-00809-1

**Published:** 2024-12-18

**Authors:** Sung-Hua Chiu, Hsiao-Chi Li, Wei-Chou Chang, Chao-Cheng Wu, Hsuan-Hwai Lin, Cheng-Hsiang Lo, Ping-Ying Chang

**Affiliations:** 1 Department of Electrical Engineering, National Taipei University of Technology, Taipei, Taiwan; 2 Department of Radiology, Tri-Service General Hospital, National Defense Medical Center, Taipei, Taiwan; 3 Division of Gastroenterology, Department of Internal Medicine, Tri-Service General Hospital, National Defense Medical Center, Taipei, Taiwan; 4 Department of Radiotherapy, Tri-Service General Hospital, National Defense Medical Center, Taipei, Taiwan; 5 Division of Hematology/Oncology, Department of Internal Medicine, Tri-Service General Hospital, National Defense Medical Center, Taipei, Taiwan

**Keywords:** Colorectal neoplasms, Liver neoplasms, Bevacizumab, Artificial intelligence, Deep learning, Neural networks, computer

## Abstract

**Background:**

To verify overall survival predictions made with residual convolutional neural network-determined morphological response (ResNet-MR) in patients with unresectable synchronous liver-only metastatic colorectal cancer (mCRC) treated with bevacizumab-based chemotherapy (BBC).

**Methods:**

A retrospective review of liver-only mCRC patients treated with BBC from December 2011 to Apr 2021 was performed. Patients who had metachronous liver metastases or received locoregional treatment before the initiation of BBC were excluded. The percentage of downstaging to curative treatment and overall survival (OS) were recorded. Two abdominal radiologists evaluated portal venous phase CT images based on the morphological criteria and divided the images into Groups 1, 2, and 3. These images were used to establish the radiologists-determined morphological response (RD-MR), which classified patients into responders and non-responders based on the morphological change 3 months after the initiation of BBC. Then, the Group 1 and 3 images classified by the radiologists were input into ResNet as the training dataset. The trained ResNet then redivided the Group 2 images into Groups 1, 2 and 3. The ResNet-MR was determined on the basis of these redivided images and the initial Group 1 and 3 images classified by the radiologists.

**Results:**

Eighty-four patients were included in this study (53 males and 31 females, with a median age of 60.0 years). The follow-up time ranged from 10 to 86 months. A total of 407 CT images were input into ResNet as the training dataset. Both RD-MR and ResNet-MR correlated with OS (*p* value = 0.0167 and 0.0225, respectively). Regarding discriminatory ability for mortality, ResNet-MR had higher area under curve than RD-MR at both 1 year and 2 years and showed a significant difference in discriminatory ability (*p-*value = 0.0321) at 2 years. RD-MR classified 28 patients (33.3%) as responders, and ResNet-MR classified an additional 16 patients (19.0%) as responders; these 16 patients had longer OS than the remaining non-responders in the RD-MR group (27.49 versus 21.20 months, *p* value = 0.043) and had a higher percentage of downstaging (37.5% versus 17.5%, *p* value = 0.1610).

**Conclusions:**

In CRC patients with liver metastases treated with BBC, prediction of survival can be improved with the aid of ResNet, enabling optimized individualized treatment.

**Supplementary Information:**

The online version contains supplementary material available at 10.1186/s40644-024-00809-1.

## Introduction

According to GLOBOCAN 2018 data [1], colorectal cancer (CRC) is the third most deadly and fourth most commonly diagnosed cancer worldwide, and approximately 30% of patients with metastatic colorectal cancer, the liver is the only affected visceral organ [2]. Bevacizumab can block the angiogenesis associated with tumor growth by inhibit VEGF-A expression [3], and bevacizumab-based chemotherapy (BBC) has shown benefit as a first-line therapy for metastatic colorectal cancer (mCRC) [4]. Because of the unique biological effects of bevacizumab, the RECIST criteria, a tumor size-based evaluation method, fail to accurately predict tumor response to BBC [5]. In contrast, the morphological criteria, which evaluates metastatic lesions based on their border and heterogenicity, was proven to have stronger associations with pathologic response and overall survival than the RECIST criteria in BBC-treated mCRC [6]. However, this predictive model is a radiologists-determined classification system with inevitable interobserver and intraobserver bias, and improvement of this model may achieve better survival prediction.

Deep learning has the ability to predict patient outcomes [7–10] with different diseases, which provides clinicians with personalized treatment decisions. A residual convolutional neural network (ResNet) is a type of neural network [11] aims to resolve the vanishing gradient problem faced by a convolutional neural network with too many layers and computational costs.

In this retrospective study, ResNet was introduced into the morphological criteria and established ResNet-determined morphological response (ResNet-MR). Then, ResNet-MR was compared to radiologists-determined morphological response (RD-MR) in the prediction of patient survival and the percentage of downstaging in mCRC patients with liver-only metastases who were treated with BBC.

## Materials and methods patient

### Population and inclusion and exclusion criteria

This study was approved by the Ethical Committee of our Institutional Review Board and a waiver of informed consent was granted for the chart and image review. The study was also in compliance with the Health Insurance Portability and Accountability Act. The diagnostic accuracy was reported in accordance with STARD 2015 guideline. The main inclusion criteria were as follows: (a) synchronous liver-only metastases in adult (20–90 years old) CRC patients who received combination therapy involving chemotherapy and target therapy with bevacizumab (with a baseline CT scan obtained within one month before initiation of treatment with bevacizumab); (b) unresectable liver metastases, which were defined as two contiguous hepatic segments that could not be preserved, vascular inflow and outflow or biliary drainage that was inadequate, or a volume of the future liver remnant that was 20% or less of the total estimated liver volume [12]; and (c) patients who had received at least two consecutive contrast-enhanced CT studies of the abdomen that contained the portal venous phase. The main exclusion criteria were as follows: (a) concomitant transarterial treatment or radiofrequency ablation (RFA) performed within 6 months before the initiation of combination therapy; (b) death or development of metastases out of the liver within 3 months after the initiation of combination therapy; (c) loss to follow-up within 3 months after the initiation of combination therapy; (d) transarterial chemoembolization or radioembolization for liver metastases after the initiation of combined therapy; (e) hepatic vein/portal vein invasion or intrahepatic duct dilatation on the initial CT scan (which has been proven to be a predictor of poor overall survival in mCRC patients with liver metastases) [13], and (f) maximal lesion size less than 1 cm in axial diameter.

**Fig. 1:**
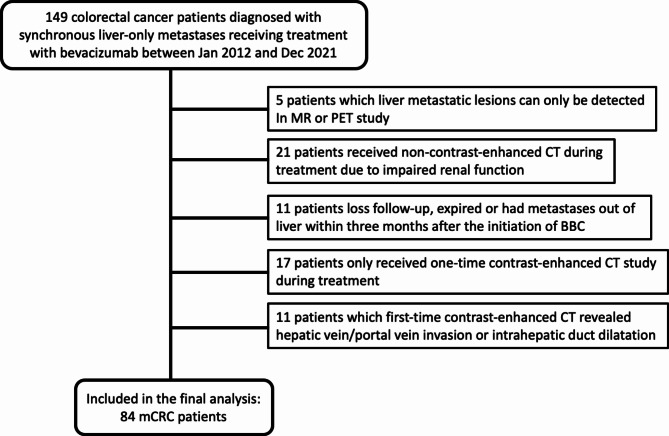
Flow chart representing the inclusion of the patients

A retrospective search of colorectal cancer databases was performed, and 84 mCRC patients treated from December 2011 to April 2021 fulfilled the above inclusion and exclusion criteria and were eligible for this study (as shown in Fig. 1). There were 53 males and 31 females, with ages ranging from 24 to 86 years old at the time of diagnosis of liver metastasis of CRC (mean age ± standard deviation: 59.5 ± 14.2 years).

### Clinical data collection

Information on demographics, location of the primary tumor, initial TNM stage, histological grade, expression of epidermal growth factor receptor (EGFR), mutation status of K-RAS, and tumor markers (including carcinoembryonic antigen and carbohydrate antigen 19 − 9) were recorded. In addition, curative management of liver metastatic lesions, including surgical resection and RFA, was also documented.

### Evaluation of treatment response by radiologists

**Fig. 2:**
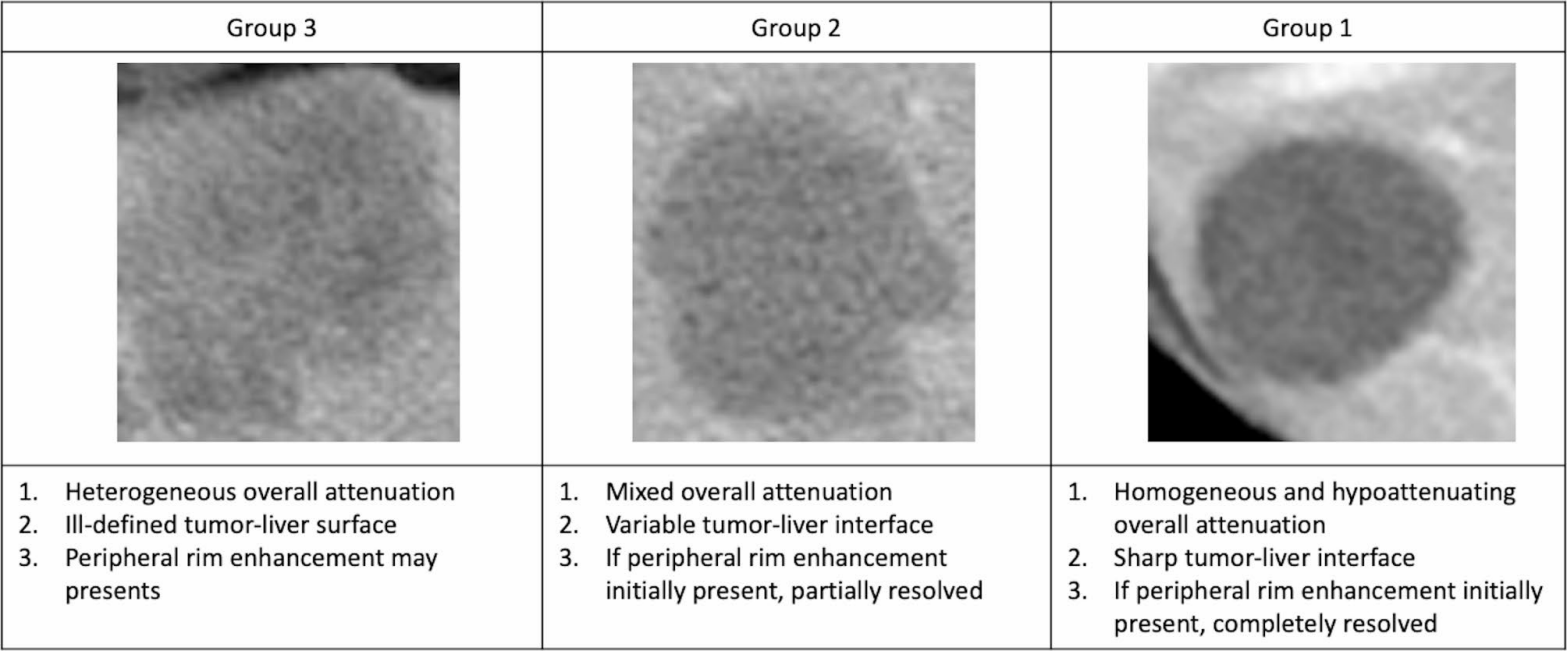
Morphological criteria [6]for evaluation of treatment response of bevacizumab-based chemotherapy

Response to treatment, as determined according to morphological criteria [6], was assessed independently by two radiologists with 8 and 18 years of experience in abdominal oncologic imaging. The radiologists were blinded to patient outcomes. Discrepancies between the radiologists were resolved by additional review and consensus. Each labeled CT scan was categorized as Group 1, 2, or 3 based on overall attenuation, tumor-liver surface, and peripheral rim of enhancement (as presented in Fig. 2). After the labeled results were recorded by each reader, the two readers met to resolve differences in readings by consensus. For each patient, in addition to baseline CT examination before BBC, at least one follow-up CT examination (with three-month interval) was received after initiation of BBC. Part of patients had an additional follow-up CT examination. All CT examinations were labelled by radiologists for ResNet training, and only first follow-up CT examinations were selected for determining responsiveness of BBC. RD-MR was used to classify patients as responders and non-responders based on the morphological change 3 months after initiation of BBC. If the metastatic lesions changed from Group 2 or 3 to Group 1 after treatment, it was classified as responders. If one did not change to Group 1 after treatment, it was classified as non-responders. In patients with multiple tumors, morphologic response was assigned based on the one metastatic lesion with the worst response. For instance, one patient had two metastatic lesions; one was classified as a responder, and the other was classified as a non-responder; in this study, this patient was classified as a non-responder. If more than one metastatic lesion had the same response, the largest lesion was selected.

### CT imaging labeling and the ResNet architecture

**Fig. 3:**
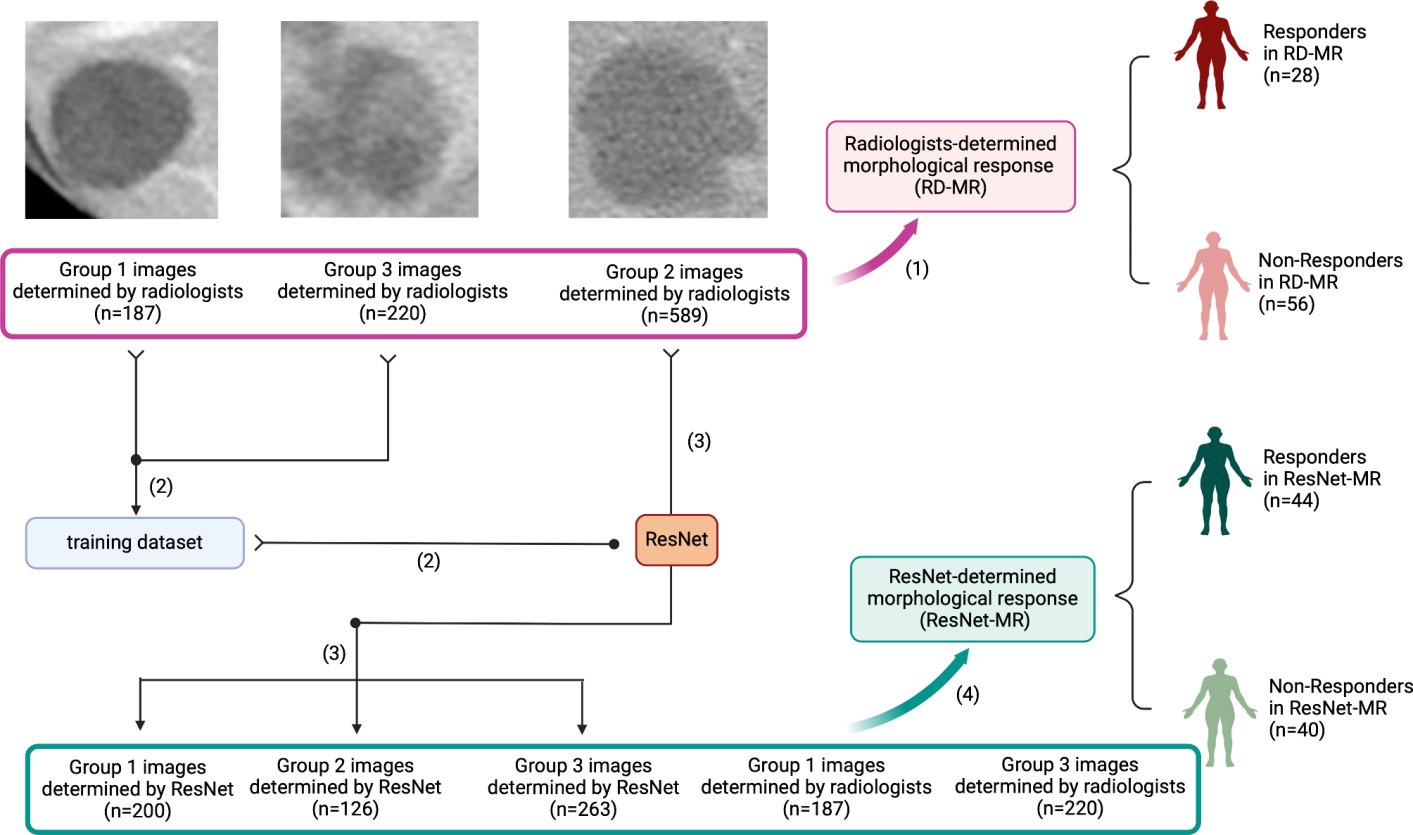
Flow chart of this study. (1) Each labeled CT scan was categorized as Group 1, 2, or 3 by radiologists and then RD-MR was established. (2) Group 1 and 3 images were inputted into ResNet as training dataset. (3) Group 2 images were then inputted into ResNet, which were be classified as Group 1, 2, or 3. (4) Finally, Group 1, 2, and 3 images classified by ResNet combined with initial Group 1 and 3 categorized by radiologists to establish ResNet-MR. Created with BioRender.com

In this study, the ResNet10 architecture was used as the basis for the network. Manual segmentation was performed by two radiologists as same as the ones who assessed morphological response. The region of interest (ROI) contained the entire volume of the metastatic lesion on the portal venous phase CT images. Labeling was performed on an open-source software platform (Labelme, a graphical image annotation tool, http://labelme.csail.mit.edu). During labeling, lesions with the following characteristics were excluded: (1) lesions encasing hepatic arteries, portal veins or hepatic veins or (2) lesions located in the subcapsular region, with part of their border contacting the peritoneal fat, diaphragm, stomach, gallbladder or kidneys rather than the normal liver parenchyma. For each CT scan, only the one metastatic lesion with the worst response and the largest size was selected for labeling. Then, the ROI was resized to 64 × 64 pixels. Group 1 and 3 CT images labelled by radiologists were input into ResNet10 as training dataset (as presented in Fig. 3).

**Fig. 4:**
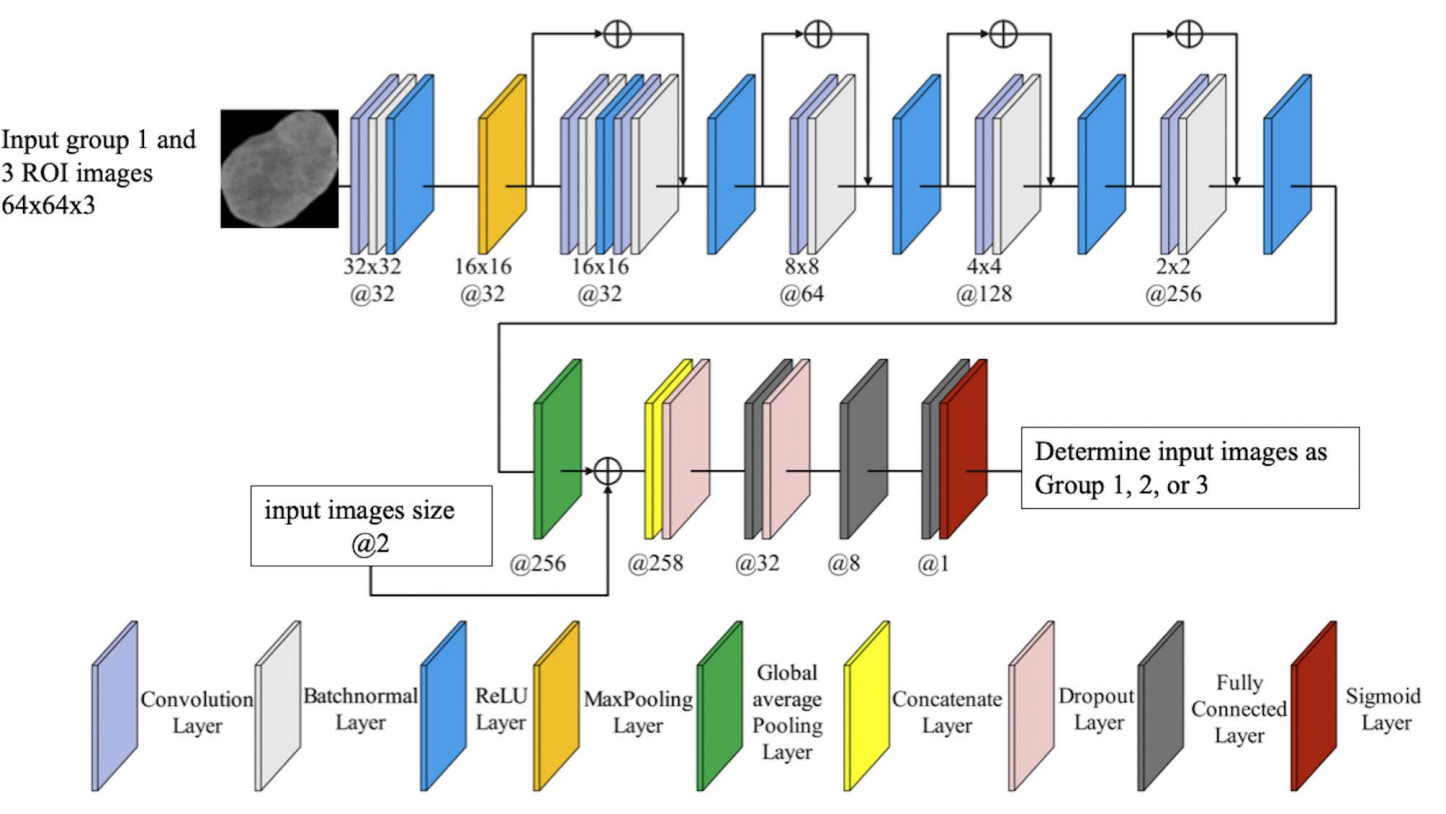
Architecture of ResNet constructed in this study

The architecture of ResNet10 and a flowchart of the deep learning approach for CT images are presented in Fig. 4. This architecture was modified and optimized based on prior study [14]. During training, the weights were optimized via Adam optimization with a batch size of 32. The learning rate and the number of epochs were set to 0.00001 and 120, respectively. Binary cross-entropy was used as the loss function. Because tumor size is an important parameter for evaluating the therapeutic response, it is also input into ResNet. The sigmoid function acted as the activation function to compute the probability before the output layer. The performance of the deep learning model was estimated by the AUC and by accuracy. After training, Group 2 CT images labelled by radiologists were input into ResNet, which were then classified as Group 1,2 or 3. Predictions of metastatic lesions were determined by the worst response among all slices. After that, ResNet-MR could be determined in accordance with the same definition of responders and non-responders in the RD-MR.

### Assessment of patient outcomes

Overall survival (OS) was calculated from the onset of BBC treatment to the date of death, with censoring for live patients performed on 30 Apr 2021. Median follow-up was calculated using OS without considering death or survival status. Downstaging was defined as patients who underwent surgical resection or RFA achieves the same overall and disease-free survival rate as surgical resection for patients with liver metastases [15].

### Statistical analysis

Statistical analyses were performed with software packages (SPSS for Windows, version 20.0; SPSS Chicago, IL), and differences were considered significant when *p* < 0.05. The results are expressed as the mean value ± standard deviation for continuous variables and as the median value, absolute frequency, and percentage for categorical variables. Differences between subgroups were evaluated by the chi-squared test or Fisher’s exact test for categorical variables and by Student’s t test for quantitative variables. Intraobserver variability was not estimated, as each radiologist assessed the CT images only once. Curve of OS were determined using the Kaplan–Meier method and compared using the log rank (Mantel‒Cox) test. The area under the curve (AUC) was calculated to evaluate discriminatory power for predicting mortality at 1-year and 2-year intervals. The last date for data collection was Apr 30, 2021.

## Results

### Patient characteristics

The baseline demographic, clinical and pathological characteristics of 84 mCRC patients are shown in Table 1. The rectum (32.1%) and sigmoid colon (32.1%) were the most common primary tumor sites. A total of 64.3% of patients had T3 disease at diagnosis, and only 17.9% of patients did not have lymph node involvement. According to the inclusion criteria of this study, all patients were stage IVA based on the AJCC 8th Edition Cancer Staging Form [16]. A total of 71.4% of patients had moderate differentiation of the primary CRC lesion. EGFR mutations were confirmed in 59.5% of patients, and 21.4% of patients had KRAS mutations. Before the initiation of combination therapy, 83.3% of patients had elevated CEA levels, and 73.8% of patients had elevated CA 19 − 9 levels. A total of 90.5% of patients received FOLFOX treatment during BBC, and the remaining patients received FOLFIRI treatment. The mean treatment duration of BBC was 8.5 ± 4.1 months. Of the included 84 patients, 85.7% had multiple metastatic lesions (as presented in Table 1). Bilobar involvement of liver metastases occurred in 72.6% of patients, and the mean maximal tumor diameter was 4.52 ± 3.12 cm. The median and IQR time between CT examinations were 99 and 22 days, respectively.

**Table 1:** Baseline demographic, clinical, pathological and radiological characteristics

Characteristics	Total (*n* = 84)
Mean age ± standard deviation (y)	59.5 ± 14.2
Median age (yr)	60.0
Gender	
Male	53 (63.1%)
Female	31 (36.9%)
Primary tumor site	
Rectum	27 (32.1%)
Sigmoid colon	27 (32.1%)
Descending colon	7 (8.3%)
Transverse colon	12 (14.3%)
Ascending colon	9 (10.7%)
Cecum	2 (2.4%)
Primary tumor T stage	
T2	13 (15.5%)
T3	54 (64.3%)
T4	17 (20.2%)
Primary tumor nodal status	
N0	15 (17.9%)
N1	37 (44.0%)
N2	32 (38.1%)
Histological grade	
Well differentiation	10 (11.9%)
Moderate differentiation	60 (71.4%)
Poor differentiation	14 (16.7%)
EGFR mutation	
Wild type	30 (35.7%)
Mutation	50 (59.5%)
Not evaluable	4 (4.8%)
KRAS	
Wild type	47 (56.0%)
Mutation	18 (21.4%)
Not evaluable	19 (22.6%)
Median CEA level (ng/ml)	59.78
Elevated CEA level	70 (83.3%)
Median CA 19 − 9 level (ng/ml)	99.53
Elevated CA 19 − 9 level	62 (73.8%)
Tumor number of liver metastases	
Solitary	12 (14.3%)
Multiple	72 (85.7%)
Unilobar/Bilobar disease	
Unilobar	23 (27.4%)
Bilobar	61 (72.6%)
Maximal tumor diameter ± standard deviation (cm)	4.52 ± 3.12

Abbreviations *CA* = Carbohydrate antigen, *CEA* = Carcinoembryonic antigen, *EGFR* = Epidermal growth factor receptor

### BBC treatment of mCRC patients

Most patients (90.5%) received FOLFOX treatment during the course of BBC (as shown in Table 2). The treatment duration of bevacizumab was 8.5 ± 4.1 months. After the use-up of bevacizumab, 55.9% of patients received BBC as maintenance treatment; the remaining patients received only chemotherapy after bevacizumab use-up. After BBC treatment, 25% of patients achieved downstaging; among these patients, 21 patients underwent hepatectomy for liver metastatic lesions, 7 patients underwent RFA, and 4 patients underwent both hepatectomy and RFA.

**Table 2:** Antitumor treatment during and after Bevacizumab-based chemotherapy

Characteristics	Total (*n*=84)
Concurrent chemotherapy	
FOLFOX	76 (90.5%)
FOLFIRI	8 (9.5%)
Treatment duration of BBC (mean± standard deviation, months)	8.5 ± 4.1
Maintenance treatment after use-up of BBC	
BBC	47 (55.9%)
FOLFOX	15 (17.9%)
FOLFIRI	9 (10.7%)
Irinotecan	4 (4.8%)
Others	9 (10.7%)
Downstaging	24 (28.6%)
Receive hepatectomy after BBC	21 (25.0%)
Receive RFA after BBC	7 (8.3%)
Receive both hepatectomy and RFA after BBC	4 (4.8%)

Abbreviations *BBC* = Bevacizumab-based chemotherapy, *RFA* = Radiofrequency ablation

### RD-MR and ResNet-MR

Regarding the 84 included patients, RD-MR classified patients into 28 responders and 56 non-responders (as shown in Fig. 3). Then, 996 CT images of liver metastatic lesions from these 84 patients were labeled by radiologists. Among the evaluated images, 187 images were labeled as Group 1, 589 images were labeled as Group 2, and 220 images were labeled as Group 3. Images of Groups 1 and 3 were input into ResNet and split into training and validation datasets at a 3:1 ratio. Then, 589 Group 2 images were input into ResNet and redivided into Groups 1,2 and 3. Based on the output of ResNet, ResNet-MR classified the patients into 44 responders and 40 non-responders.

### Differences in responders between RD-MR and ResNet-MR

**Fig. 5:**
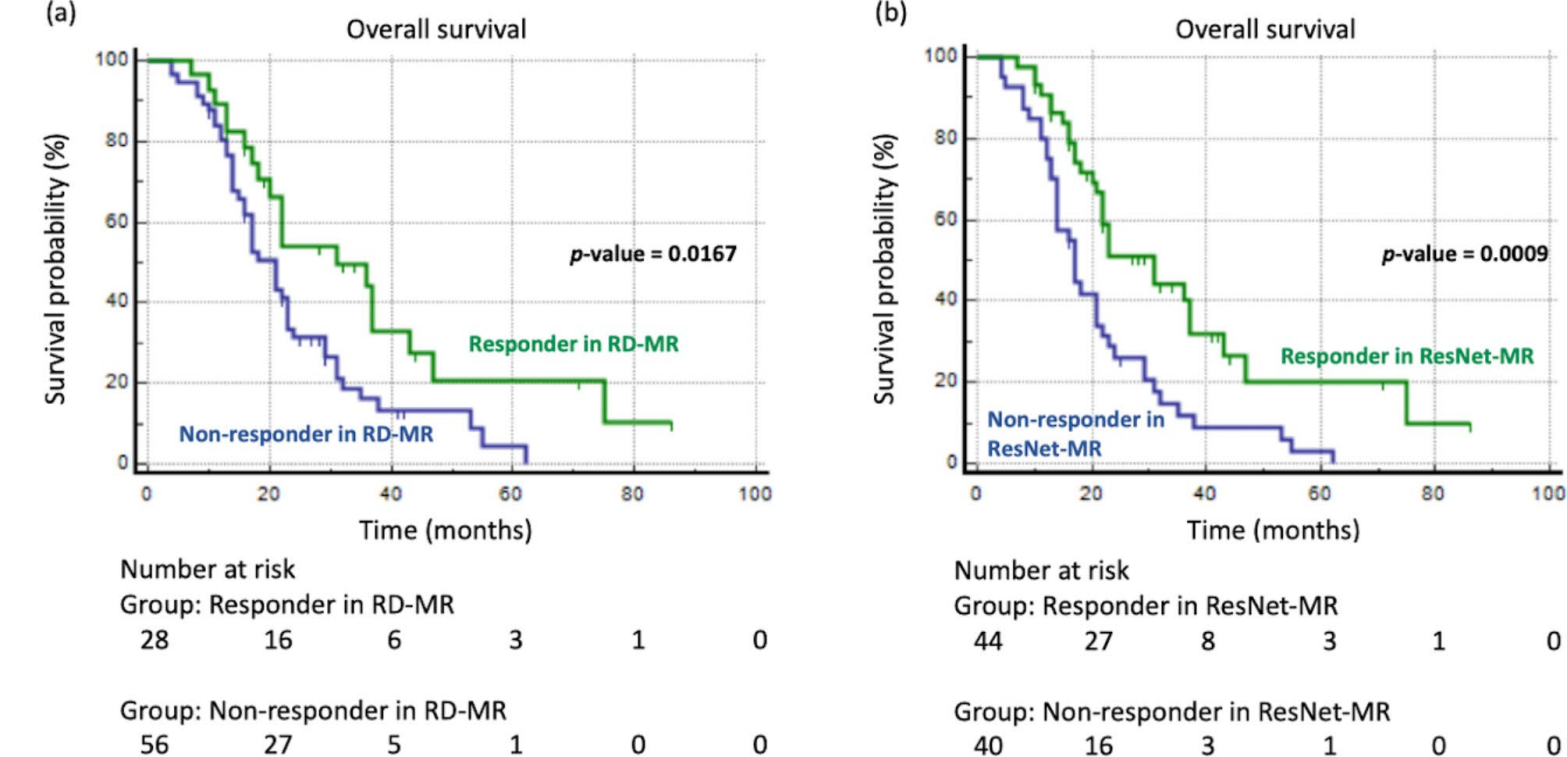
Kaplan Meier overall survival curves in responders and non-responders in (**A**) RD-MR and (**B**) ResNet-MR

Both RD-MR and ResNet-MR correlate with overall survival in mCRC patients. Regarding RD-MR, the OS of responders and non-responders was 37.09 months and 23.91 months, respectively (*p-*value = 0.017, Fig. 5a). With ResNet-MR, the OS of responders and non-responders was 36.51 months and 21.20 months, respectively (*p-*value < 0.001, Fig. 5b). With regard to receiving curative treatment of hepatic metastatic lesions after bevacizumab-based chemotherapy, there was no difference in responders between RD-MR (39.3%) and ResNet-MR (38.6%). Regarding discriminatory ability for mortality at 1 and 2 years (as shown in Table 3), as evaluated by ROC curve area analysis, ResNet-MR had higher AUC than RD-MR at both 1 year and 2 years; in addition, at 2 years, ResNet-MR showed a significant difference in discriminatory ability (*p-*value = 0.0321). RD-MR did not reach a significant difference in the discriminatory ability for mortality at either 1 year or 2 years.

**Table 3:** Discriminatory ability for mortality at 1 year and 2 years, by RD-MR and ResNet-MR

	1 year survival			2 years survival		
	AUC	95% CI	*p*-value	AUC	95% CI	*p*-value
RD-MR	0.546	0.433–0.656	0.2496	0.609	0.492–0.718	0.0696
ResNet-MR	0.578	0.465–0.686	0.0573	0.615	0.498–0.723	0.0321*

Abbreviations *AUC* = Area under curve, *CI* = Confidence level

### “New-responders” in ResNet-MR

**Fig. 6:**
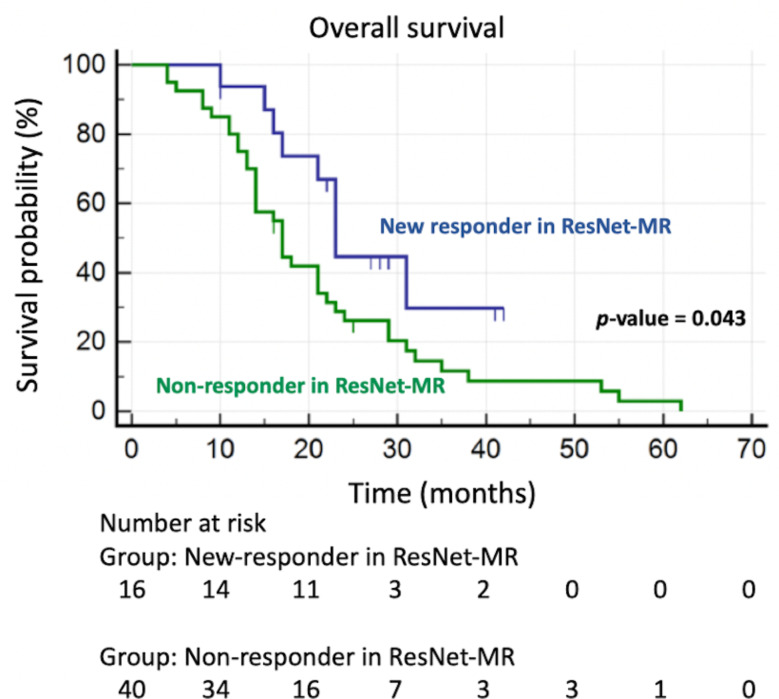
Kaplan Meier overall survival curves in “new-responders” (originally determined as non-responders in RD-MR, and re-classified as responders in ResNet-MR) and non-responders in ResNet-MR

Sixteen patients classified as responders in ResNet-MR were initially determined to be non-responders in RD-MR; in addition, all non-responders in ResNet-MR were also determined to be non-responders in RD-MR. These 16 patients (who were labeled as “new-responders” in this study) had longer OS than.

non-responders in ResNet-MR (27.49 months versus 21.20 months, *p-*value = 0.043) (as shown in Fig. 6). In addition, among these new-responders, 37.5% of them received curative treatment of hepatic metastatic lesions, compared with 17.5% of non-responders in ResNet-MR (although no statistically significant differences were achieved with *p-*value = 0.1610).

## Discussion

In this study, a novel application of ResNet in predicting the therapeutic response to BBC in colorectal cancer patients with synchronous liver-only metastases was demonstrated. ResNet showed the ability to predict patient survival and identify patients who were responsive to BBC but were classified as non-responsive by radiologists; these patients had longer OS and a higher chance of receiving a curative treatment of hepatic metastatic lesions than non-responders.

**Fig. 7:**
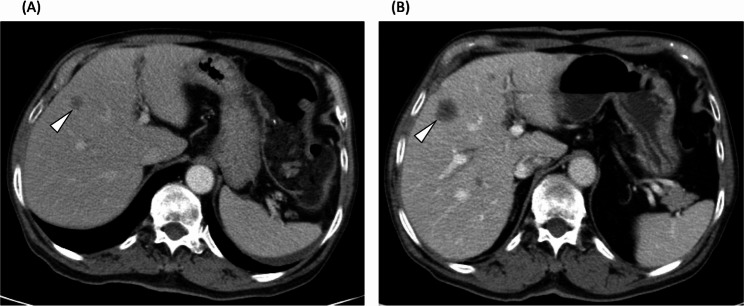
A 72-year-old with moderately-differentiated adenocarcinoma of sigmoid colon with liver metastasis. Before initiation of bevacizumab-based chemotherapy (**A**), CT scan showed a 1.1-cm metastatic lesion (arrowhead) in the segment 5/8 of liver. After three months of treatment with bevacizumab-based chemotherapy, CT scan (**B**) demonstrated increased size (2.2 cm) of this metastatic lesion (arrowhead) with indistinct liver-tumor surface. RD-MR defined this patient as a non-responder. In ResNet-MR, this patient was classified as a responder. The patient survived for 28 months after initiation of bevacizumab-based chemotherapy and was alive on the date of final censoring

**Fig. 8:**
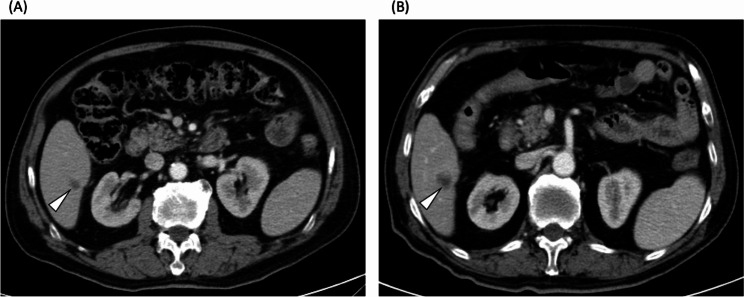
A 86-year-old with well-differentiated adenocarcinoma of sigmoid colon with liver metastasis. Before initiation of bevacizumab-based chemotherapy (**A**), CT scan showed a 1.1-cm metastatic lesion (arrowhead) in the segment 6 of liver. After three months of treatment with bevacizumab-based chemotherapy, CT scan (**B**) demonstrated increased size (1.6 cm) of this metastatic lesion (arrowhead) with indistinct liver-tumor surface. RD-MR defined this patient as a non-responder. In ResNet-MR, this patient was classified as a responder. The patient survived for 31 months after initiation of bevacizumab-based chemotherapy

The morphological criteria, which can be used to evaluate the tumor response based on overall attenuation, the tumor-normal liver interface, and the presence of peripheral rim enhancement, have been proven to have a significant association with overall survival in colorectal patients with liver metastases receiving BBC [6]. Homogeneous attenuation of hepatic metastatic lesions indicates responsiveness to treatment, which reflects the fibroconnective tissue within the tumor [17]. A clear tumor-normal liver interface has been shown to correlate with pathological response (the proportion of tumor cells remaining after chemotherapy) and disease-free survival [18]. Peripheral rim enhancement of hepatic metastases is mainly caused by desmoplastic reactions, inflammation and vascular proliferation [19]. Based on these three imaging parameters, morphological criteria can successfully predict the overall survival of mCRC patients. However, subjective bias related to the morphological criteria, especially the determination of Group 2 morphology (as shown in Figs. 7 and 8), will interfere with the discrimination of responders and non-responders, leading to inaccurate prognostic prediction.

To date, ResNet has been introduced into medical image analysis in different applications [20–22]. Gholizadeh-Ansari et al. [20]. showed that ResNet can improve the quality of low-dose CT images by denoising. Garg et al. [21]. performed the diagnosis of COVID-19 with CT scan under the assistance of ResNet with 94.9% of validation accuracy. ResNet had also been used in the prediction of local tumor response of hepatocellular carcinoma receiving transarterial chemoembolization, with accuracy of 84.3% [22]. For mCRC, DL has shown its ability to predict treatment response and patient survival [23, 24]. Zhu et al. [23] demonstrated that the MRI-based deep learning model predicted the pathological tumor response to preoperative chemotherapy in mCRC patients. Lu et al. [24] showed that the DL network with CT images can predict the early treatment response to chemotherapy in mCRC patients. For these two studies, Zhu et al. [23] included mCRC patients treated with standard preoperative chemotherapy (FOLFOX/FOLFIRI/XELOX) with or without bevacizumab; Lu et al. [24] collected patients from the VELOUR trial [25], which included mCRC patients treated with FOLFIRI with or without aflibercept (another antiangiogenic agent). Both studies [23, 24] included mCRC patients who were not treated with antiangiogenic agents, which have distinct antitumor effects compared with cytotoxic chemoagents. In this study, only mCRC patients treated with were enrolled, which reduced the heterogenicity caused by different therapeutic regimen.

In mCRC patients with unresectable liver metastases, the combination of bevacizumab with FOLFOX/FOLFIRI results in increases in survival, progression-free survival and response rate. For these patients, the therapeutic goal is to achieve downgrading to receive surgery or RFA, which are considered curative treatments for hepatic metastatic lesions [26]. Predicting the therapeutic response after the initiation of treatment is critical because if treatment is considered to fail, changes of the therapeutic regimen should be considered. The ResNet-MR proposed in this study has the potential to predict the therapeutic response in the first three months after the initiation of BBC, which may benefit personalized early treatment-related decision-making.

This study has some limitations. First, ResNet was trained with Group 1 and 3 images determined by radiologists, and human classification may lead to subjective bias. Second, in patients with multiple liver metastatic lesions, only the one lesion with the worst response and largest size was selected for labeling; this selection was performed by radiologists with inevitable subjective bias. Third, the ROIs were drawn manually by radiologists in this study, and the border segmentation may differ between different observers. This bias may be overcome by whole-liver segmentation in future studies. Fourth, this study was a single-center retrospective study, with small sample size and heterogeneity of the population; further prospective multi-center study may be needed for external validation of the proposed model.

In conclusion, ResNet showed its ability to predict therapeutic effect of BBC in mCRC patients, including overall survival and chance to reach downstaging, which will optimize individualized cancer treatment.

## Electronic supplementary material

Below is the link to the electronic supplementary material.

Supplementary Material 1

## Data Availability

No datasets were generated or analysed during the current study.
